# Comparative secretome analysis of *Staphylococcus aureus* strains with different within-herd intramammary infection prevalence

**DOI:** 10.1080/21505594.2021.2024014

**Published:** 2022-01-14

**Authors:** M. Filippa Addis, Salvatore Pisanu, Valentina Monistero, Alessandra Gazzola, Martina Penati, Joel Filipe, Susanna Di Mauro, Paola Cremonesi, Bianca Castiglioni, Paolo Moroni, Daniela Pagnozzi, Sebastiana Tola, Renata Piccinini

**Affiliations:** a Dipartimento Di Medicina Veterinaria, Università Degli Studi Di Milano, Lodi, Italy; b Porto Conte Ricerche, Tramariglio, Alghero, Italy; c Institute of Agricultural Biology and Biotechnology, National Research Council, Lodi, Italy; d Quality Milk Production Services, Animal Health Diagnostic Center, Cornell University, Ithaca, NY, USA; e Istituto Zooprofilattico Sperimentale Della Sardegna “G. Pegreffi”, Sassari, Italy

**Keywords:** Proteomics, mammary gland, dairy cow, mastitis, intramammary infection, immune evasion, inflammation, bacterial virulence

## Abstract

*Staphylococcus aureus* is a major pathogen causing intramammary infection and mastitis in dairy cows. *S. aureus* genotypes (GT) can differ significantly in their ability to diffuse and persist in the herd; while the association of virulence gene carriage with epidemiological behavior remains unclear, a role for secreted proteins has been postulated. We characterized the secretome of six *S. aureus* strains belonging to two genotypes with opposite within-herd prevalence, GTB (high) and GTS (low), corresponding to sequence types (ST) 8 and 398, by high-resolution tandem mass spectrometry and differential analysis with Proteome Discoverer. Data are available via ProteomeXchange with identifier PXD029571. Out of 720 identified proteins, 98 were unique or more abundant in GTB/ST8 and 68 in GTS/ST398. GTB/ST8 released more immunoglobulin-binding proteins, complement and antimicrobial peptide inhibitors, enterotoxins, and metabolic enzymes, while GTS/ST398 released more leukocidins, hemolysins, lipases, and peptidases. Furthermore, GTB/ST8 released the von Willebrand factor protein, staphylokinase, and clumping factor B, while GTS released the staphylococcal coagulase and clumping factor A. Hence, GTB/ST8 secretomes indicated a higher propensity for immune evasion and chronicity and GTS/ST398 secretomes for cellular damage and inflammation, consistent with their epidemiological characteristics. Accordingly, GTS/ST398 secretions were significantly more cytotoxic against bovine PBMCs *in vitro*. Our findings confirm the crucial role of extracellular virulence factors in *S. aureus* pathogenesis and highlight the need to investigate their differential release adding to gene carriage for a better understanding of the relationship of *S. aureus* genotypes with epidemiological behavior and, possibly, disease severity.

## Introduction

*Staphylococcus aureus* is still a relevant cause of bovine mastitis worldwide, despite efforts to control its presence and diffusion in dairy herds. A multitude of factors associated with the phenotypic and genotypic characteristics of the infecting strain influence its ability to spread and persist in the herd, as well as the outcome of disease. *S. aureus* strains isolated from milk samples of cows with mastitis are genetically heterogeneous and can harbor an extensive array of virulence-associated genes. However, only a few genotypes have been linked to a high within-herd prevalence of intramammary infection (IMI) [1–3]. Previous European studies used Ribosomal Spacer PCR (RS-PCR) to classify *S. aureus* strains in genotypes. These demonstrated that *S. aureus* belonging to genotype B (GTB) are associated with a high diffusion within the herd and are frequently isolated from dairy farms in Central Europe and in Italy [4]. Genotype B generally corresponds to Sequence Type 8 (ST8), a bovine-adapted genotype originated from a human-to-cow host jump [5]. The highly contagious *S. aureus* GTB/ST8 strains are characterized by the presence of specific genes coding for enterotoxins (*sea, sed*, and *sej*) [1–3,6,7]. On the other hand, *S. aureus* belonging to genotype S (GTS), which generally corresponds to ST398, are more likely associated with sporadic IMI [2–4,6]. *S. aureus* ST398 have a broad host range and represent a threat to public health for their ability to affect livestock, especially swine, and humans [8], and to acquire multidrug resistance; methicillin-resistant *S. aureus* strains frequently belong to GTS/ST398 [6]. In 2017, Capra and coworkers [9] performed a detailed genomic and transcriptomic investigation of three *S. aureus* GTB/ST8 and three GTS/ST398 strains to shed further light on their differential characteristics. Their work revealed relevant differences in several genes associated with virulence factors, with some of them being exclusive of one genotype.

*S. aureus* can produce diverse secreted and surface-associated virulence factors that contribute collectively to colonization and invasion of host cells and tissues, as well as evasion of immune responses [10]. Wall-bound virulence factors include Microbial Surface Components Recognizing Adhesive Matrix Molecules (MSCRAMMs) that mediate adherence to different substrates of the host [10,11], as well as Secreted Expanded Repertoire Adhesive Molecules [SERAMs). Among other findings, Capra and coworkers [9] observed a high polymorphism in the *fnbB* gene between *S. aureus* GTB/ST8 and GTS/ST398, resulting in a truncated form of the protein in the latter strains and possibly affecting *S. aureus* colonization and infection efficiency [9, 12]. In addition, the staphylococcal complement inhibitor (SCIN), which helps the bacteria to survive in the host by preventing chemotaxis and phagocytosis [13], was over-expressed in GTB/ST8 and down-regulated in GTS/ST398 [9]. GTB/ST8 strains also showed higher expression of signal transduction Target of RNAIII Activating Protein (TRAP), which activates RNAIII synthesis increasing the pathogenic potential of the bacteria [14,15]. Adding to differential secretion or release, major differences in the composition of *S. aureus* secretome are related to differences in transcriptional regulation by the *agr* system, resulting in the expression of diverse secreted virulence factors [16,17].

Genomic and transcriptomic investigations, however, may not reflect the actual composition of secreted virulence factors [18] which can be better understood with the analysis of the extracellular proteome (secretome) [16]. Previous studies found that *S. aureus* may differ considerably in the composition and abundance of secreted proteins [16,18]. Importantly, these are also thought to represent the main reservoir of virulence factors, and grouping of clinical isolates based on their secretome profile can be related to virulence [19]. Investigating the bacterial secretome by high-performance shotgun proteomics is therefore a powerful approach for exploring staphylococcal pathogenicity and developing novel strategies for *S. aureus* detection and control, including vaccine design [17,20].

Here, we report the detailed characterization of the secretome of the three *S. aureus* GTB/ST8 and three GTS/ST398 strains previously investigated by genomics and transcriptomics [9] and associated with high within-herd *vs* low within-herd prevalence, respectively. Some relevant phenotypic traits related to the secretome differences are also presented.

## Materials and methods

### S. aureus *strains, culture conditions, and growth analysis*

This study was carried out on 6 *S. aureus* strains. These had been isolated from the milk of cows with subclinical mastitis belonging to herds with different IMI prevalence and characterized by Ribosomal Spacer polymerase chain reaction (RS-PCR) and Multilocus sequence typing (MLST) [6,9]. Three strains, identified as GTS/ST398, had been isolated from herds with low IMI prevalence (range: 2–4%), and three strains, identified as GTB/ST8, had been isolated from herds with high IMI prevalence (range: 49–62%) [6]. For this study, each *S. aureus* strain was thawed and revitalized in Brain Heart Infusion broth (BHI, Oxoid, Rodano, IT) overnight at 37°C. After incubation, the optical density of each suspension was measured at 620 nm (OD_620_) using a SpectraMax 340PC spectrophotometer (Molecular Devices, LLC, CA), diluted to an OD_620_ value of 0.08–0.1 in BHI broth and incubated overnight at 37°C. For whey preparation, fresh milk from 10 single quarters of as many cows with SCC ≤7,000/ml was ultracentrifuged twice for 30 min at 45,000 g at 4°C. The supernatant was transferred to a sterile bottle and then sterilized through a 0.22 μm Millipore filter. Revitalized cultures were used for inoculating milk whey with the same dilutions calculated for BHI and incubated overnight at 37°C. On the following day, overnight culture suspensions obtained in both media were diluted 1:100 in the corresponding medium (BHI or milk whey, respectively) and incubated with agitation at 37°C for 7 hours. Bacterial growth was evaluated by plating each suspension on Blood agar plates (Oxoid) in triplicate at different time points during liquid culture for evaluating the colony forming units (CFU) per mL. Statistical analysis was carried out on the growth curves with GraphPad Prism 9 (San Diego, CA) using the paired t-test for means.

### Preparation of secreted proteins

*S. aureus* suspensions for proteomic analysis and cell viability assays were prepared in BHI broth by seeding with the overnight culture suspensions obtained as described above and incubating at 37°C with agitation for 3.5 h. Then, bacterial cultures were centrifuged at 9,300 g for 5 minutes and supernatants were transferred to new sterile Eppendorf tubes. For SDS-PAGE and mass spectrometry analysis, bacterial culture supernatants were concentrated 10x in Amicon Ultra-0.5 centrifugal filter units with Ultracel-10 membrane (Millipore, Billerica, MA, USA). Protein concentration was evaluated with the Pierce™ 660 nm Protein Assay Kit (Thermo Scientific, San Jose, CA, USA).

### SDS-PAGE analysis

SDS-PAGE separation of proteins was carried out on a Tetra Cell™ with AnykD™ precast gels (Bio-Rad Laboratories, Hercules, CA, USA) according to the user manual. The concentrated BHI supernatants were mixed with loading buffer, reduced and denatured, loaded into the wells, and subjected to electrophoretic separation as described previously [21]. After the run, the gels were stained with Coomassie SafeStain (Bio-Rad) for protein visualization.

### Protein digestion and peptide quantitation

For shotgun proteomic analysis, the concentrated supernatants were processed by filter-aided sample preparation (FASP) [22]. Briefly, samples were subjected to reduction, alkylation, and trypsin digestion within Amicon Ultra-0.5 centrifugal filter units with a 3 kDa cutoff membrane. Peptide concentrations were measured by absorbance at 280 nm using a NanoDrop 2000 spectrophotometer (Thermo Scientific, San Jose, CA, USA) using MassPREP *E. coli* Digest Standard (Waters, Milford, MA, USA) for calibration.

### Tandem mass spectrometry analysis of peptides

Peptide mixtures were subjected to tandem mass spectrometry (MS/MS) analysis on a Q-Exactive coupled with an UltiMate 3000 RSLCnanoLC system (Thermo Scientific, San Jose, CA, USA). Peptide mixtures (4 μg) were concentrated and washed in a trapping precolumn (Acclaim PepMap C18, 75 μm × 2 cm nanoViper, 3 μm, 100 Å, Thermo Scientific) and then fractionated on a C18 RP column (Acclaim PepMap RSLC C18, 75 μm × 50 cm nanoViper, 2 μm, 100 Å, Thermo Scientific) at flow rate of 250 nL/min. The linear gradient lasted 245 minutes from 5 to 37.5% eluent B (0.1% formic acid in 80% acetonitrile) in eluent A (0.1% formic acid). Fragmentation was done by Higher Energy Collisional Dissociation (HCD) with nitrogen as the collision gas. Each sample was obtained in one growth experiment and was processed in duplicate MS/MS runs.

### Proteomic data analysis

Protein identification was carried out with Proteome Discoverer (version 2.4; Thermo Scientific) and Sequest-HT as search engine. Analysis of MS/MS spectra was carried out with the following settings. Database: *Staphylococcus aureus* (137,957 sequences retrieved from UniProt Knowledgebase (UniprotKb), release 2021_02); enzyme: trypsin, with two missed cleavages allowed; precursor mass tolerance: 10 ppm; MS/MS tolerance: 0.02 Da; charge states: +2, +3, and +4; cysteine carbamidomethylation as static modification and methionine oxidation and acetylation (Acetyl), loss of Methionine (Met-loss) and loss of Methionine-loss+Acetylation (Met-loss+Acetyl) on N-Terminal as dynamic modifications. Protein significance and peptide validation (false discovery rate, FDR, <1%) were defined with the percolator algorithm. Peptide and protein grouping were allowed according to the Proteome Discoverer algorithm by applying the strict maximum parsimony principle. A Consensus step was performed by creating two experimental groups represented by three samples for each group, processed in duplicate MS/MS runs. A standard consensus workflow was set on Proteome Discoverer 2.4 to evaluate label-free (LFQ) and precursor ion quantification. Precursor ion abundances were calculated using intensity as abundance parameter, normalized for evaluating the abundance ratio significance among the proteins identified in the two different experimental groups. The fold ratio was calculated by the pair wise ratio-based method and the maximum allowed fold ratio was set to 100. The abundance ratio (AR) was log-transformed (Log_2_) and differential proteins were predicted using the t-test (background based) and adjusting the p-value by Benjamini-Hochberg correction. Proteins were considered as significantly different if they had an abundance ratio of less than or equal to −1.5 and greater than or equal to +1.5 (−1.5≤ AR≥+1.5) with an adjusted p-value ≤0.05. Principal component analysis (PCA) was performed using the normalized abundance of all identified proteins with Proteome Discoverer 2.4. Biological processes and molecular functions of the differential proteins were retrieved from their UniProtKB entry pages. Normalized protein abundance values were calculated with Proteome Discoverer. The differential protein abundance heatmaps were prepared using Microsoft Excel^TM^.

### Isolation of PBMCs

Whole blood from 6 clinically healthy pluriparous dairy cows was collected in sterile tubes with EDTA as an anticoagulant during routine slaughtering procedures. The peripheral blood mononuclear cells (PBMCs) were purified as previously reported from 60 mL of whole blood [23]. Briefly, the tubes were centrifuged at 1,260 g for 30 min at 18°C without brake, and the buffy coat was collected and diluted in cold PBS + 2 mM EDTA (1:2 dilution). The diluted buffy coat (10 mL) was layered on 3 mL of Ficoll-Paque Plus (1.077 g/mL) and centrifuged at 1,700 g for 30 min at 4°C without brake. The PBMC ring was collected, and the cells were counted with an automatic cell counter (TC20^TM^, BioRad) in Trypan blue. The cells were then resuspended in complete medium (RPMI-1640 + 25 mM Hepes, 10% heat-inactivated FBS, 1% penicillin/streptomycin, 1% non-essential amino acids).

### Determination of cell viability

Cell viability was determined using the Cell Proliferation Kit I (MTT, Roche), following the manufacturer’s instructions. To determine the cytotoxicity of GTB/ST8 and GTS/ST398 *S. aureus* secretomes, 1 × 10^5^ cells/well were seeded in 96-well plates and incubated for 18 hours with increasing concentrations (0.5%, 1%, 2.5% and 10%) of the 1X secreted protein preparation obtained as described above but using RPMI-1640 as the growth medium. Cells incubated with complete medium only were used as control. After the incubation period, 10 μl of MTT reagent was added to each well and incubated for 4 hours; 100 μL of solubilization buffer was added and the cells were incubated overnight. Absorbance was subsequently measured with a LabSystems Multiskan plate reader spectrophotometer (LabX, Midland, Canada) at a test wavelength of 550 nm. Data were expressed as fold change compared to the control. Six technical replicates (six replicate wells) were performed for each measurement. Statistical analysis was carried out using GraphPad Prism 9 and the normal distribution of the dataset was assessed using the Shapiro Wilk test. Kruskal-Wallis and Dunn’s multiple comparisons test were used. Statistical significance was accepted at P ≤ 0.05.

## Results

### Phenotypic characteristics

**Figure 1.**
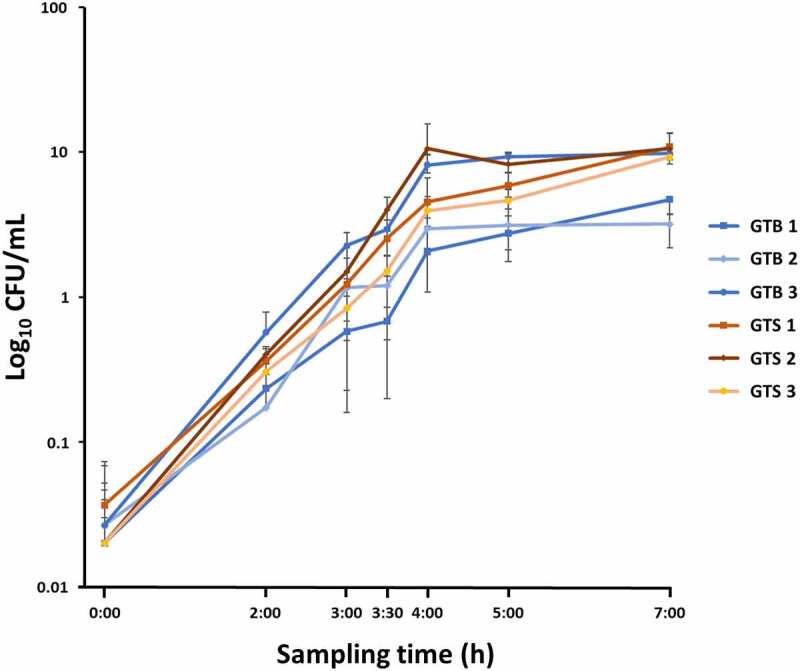
Growth curves in brain-heart infusion broth (BHI) medium of the six *Staphylococcus aureus* GTB/ST8 (shades of blue) and GTS/ST398 (shades of Orange) strains used in this study. The curves report the bacterial colony forming units (Log_10_ CFU)/mL as a function of time. Each point represents the mean (symbol) and standard deviation (bars) of three replicate CFU measurements. The X axis indicates the sampling times.

All the strains had similar morphology when plated on blood agar; the colonies were pale yellow, round, smooth, and surrounded by a halo of hemolysis. All the GTS/ST398 strain produced a β-hemolysis *versus* only one GTB/ST8 (GTB3) strain. When grown in milk whey, all GTB/ST8 strains yielded a visible, large white clot, while the GTS/ST398 did not induce protein coagulation. All strains reached the logarithmic phase of growth at 3.5 h of liquid culture in both BHI and milk whey. While GTB strains grew significantly slower than GTS strains in milk whey (p < 0.01, data not shown), no statistically significant differences were observed in BHI, the growth medium used for proteomic analysis. The growth curves observed in BHI for the six analyzed strains are illustrated in Figure 1.

### SDS-PAGE analysis of cellular and extracellular proteins

**Figure 2.**
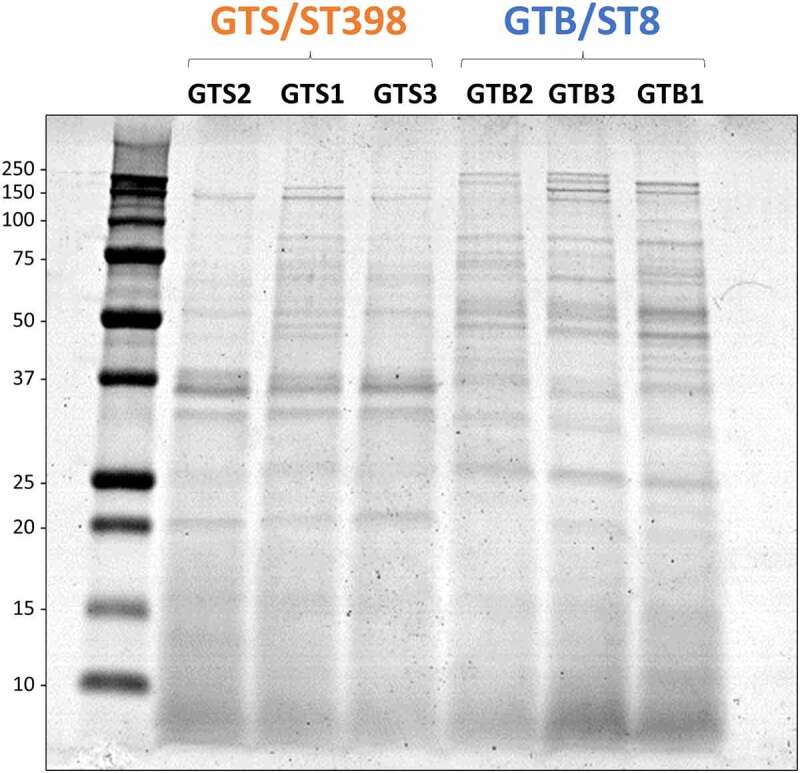
SDS-PAGE profile of the proteins secreted in brain-heart infusion (BHI) broth by the six *Staphylococcus aureus* strains evaluated in this study. The GT/ST is indicated above the name of respective *S. aureus* strains. Protein load is 10 µg per lane.

Secreted bacterial proteins were analyzed at 3.5 h of growth in BHI. The secreted proteins of the six investigated strains were first analyzed by SDS-PAGE for a visual comparison. As shown in Figure 2, secreted protein profiles showed evident differences between strains of the two genotypes. This prompted us to further investigate the secretome by high-performance differential shotgun proteomics to shed light on these differences.

### Differential shotgun proteomics; general results

**Figure 3.**
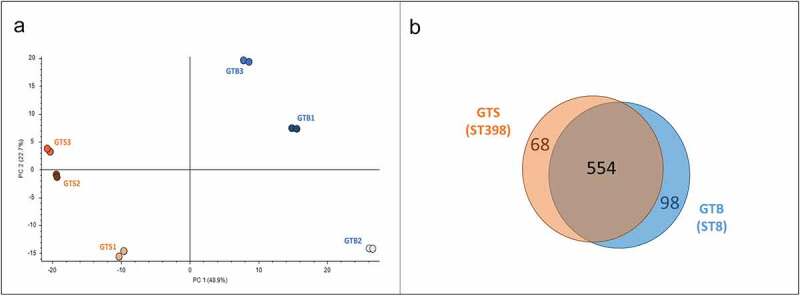
General results of the differential shotgun proteomics of the *Staphylococcus aureus* secretome obtained in brain-heart infusion (BHI) broth. (a) Principal Component Analysis based on the normalized protein abundances. (b) Venn diagram illustrating the distribution of the 720 proteins identified in the secretomes of the two GT (ST), showing shared proteins and differential proteins identified for each sample group.

By applying high-performance tandem mass spectrometry and Proteome Discoverer analysis for protein identification, we identified a total of 720 unique proteins in the six secreted protein samples. Principal component analysis (PCA) carried out on the protein normalized abundance data clustered the six strains according to the GT/ST by the first component (48.9%) (Figure 3(a)). Upon differential proteomic analysis with Proteome Discoverer, 166 proteins showed a significantly different abundance between the two genotypes: 98 were more abundant in GTB/ST8 and 68 in GTS/ST398 (Figure 3(b)).

### Functional analysis of the differential proteins

**Figure 4.**
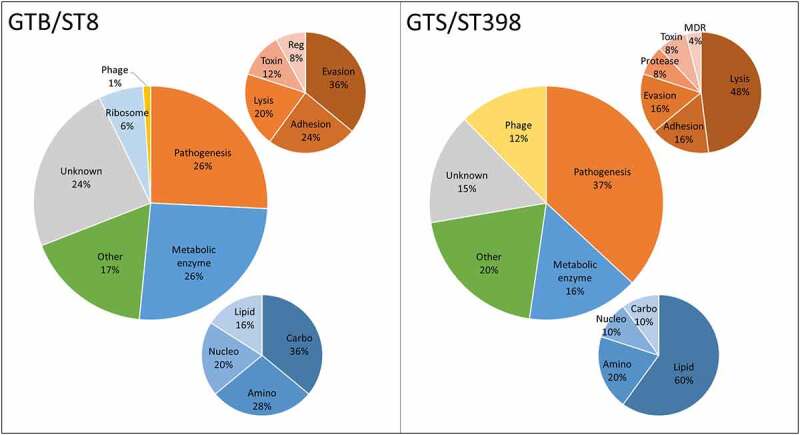
Distribution of the differential functions of the proteins secreted in brain-heart infusion (BHI) broth by the six *Staphylococcus aureus* strains evaluated in this study, classified according to the respective GT/ST. The smaller graphs illustrate the relative composition of the categories “Pathogenesis” (Orange) and “Metabolic enzyme” (blue). Abbreviations: Reg: regulation of gene expression; MDR: multidrug resistance; Amino: aminoacid metabolism; Carbo: carbohydrate metabolism; Lipid: lipid metabolism; Nucleo: nucleotide metabolism.

The differential secreted proteins identified in the GTB/ST8 and GTS/ST398 strains are reported in Tables 1 and 2, respectively. Figure 4 reports the distribution of the protein functions based on the categories reported in Tables 1 and 2. Most of the unique/differential proteins in GTS/ST398 had a well-recognized role in staphylococcal pathogenesis (37%) against 26% in GTB/ST8. In GTS/ST398, 48% mediated eukaryotic cell lysis, while in GTB/ST8 36% were primarily involved in immune evasion. Conversely, a large part of the differential proteins in GTB/ST8 (26%) were metabolic enzymes, mainly of the carbohydrate metabolism. Differential metabolic enzymes were only 16% of the GTS/ST398 secretome and 60% them belonged to the lipid metabolism. Phage-encoded proteins were 12% in GTS/ST398 and only 1% in GTB/ST8. Of note, 6% of all GTB/ST8 differential proteins were ribosomal proteins, found only in this genotype.

**Table 1.** Lists of the 98 proteins found exclusively (log_2_ + 6.64) or at significantly higher levels (log_2_ > +1.50) in the secretome of GTB/ST8 strains grown in brain-heart infusion (BHI) broth. The information reported in the first three columns (accession number, protein name, and abundance ratio – AR – expressed as the log_2_ value) was obtained by Proteome Discoverer Analysis. The last column reports biological process and molecular function information as found in the UniProtKB. The definition “other” indicates that the protein belongs to metabolic pathways different than amino acid, carbohydrate, lipid, or nucleotide metabolism

Accession	Description	AR (log_2_)	Process/function
	*Proteins detected only in GTB/ST8 secretomes*		
P21222	30 kDa neutral phosphatase	6.64	Pathogenesis/Immune evasion
A0A6H3XEA2	30S ribosomal protein S1	6.64	Ribosome
A0A7H4EEQ2	50S ribosomal protein L3	6.64	Ribosome
T1YD59	7,8-dihydro-8-oxoguanine-triphosphatase	6.64	Other
UPI001313628C	Acetyltransferase GNAT family	6.64	Other
A0A6H4H0R0	Alcohol dehydrogenase	6.64	Other
A0A2S6D6E3	Antibacterial protein	6.64	Pathogenesis/Cytolysis
A0A0E0VMF7	Argininosuccinate synthase	6.64	Amino acid metabolism
A0A7H3TGM9	CHAP domain-containing protein	6.64	Unknown/undefined
A0A6M4IG30	Class I SAM-dependent rRNA methyltransferase	6.64	Nucleotide metabolism
A0A6B5L1Z6	Dihydrolipoyllysine-residue succinyltransferase component of 2-oxoglutarate dehydrogenase complex	6.64	Lipid metabolism
A0A641A3Z7	dTMP kinase	6.64	Nucleotide metabolism
A0A6A8FZT6	DUF1292 domain-containing protein	6.64	Unknown/undefined
D0EMB3	Enterotoxin SEA	6.64	Pathogenesis/Toxin
A0A6H4VKP7	Fibronectin-binding protein A	6.64	Pathogenesis/Adhesion
A0A7H3H9A0	Formate dehydrogenase subunit alpha	6.64	Other
A0A7H1UCG3	Formyl peptide receptor-like 1 inhibitory protein	6.64	Pathogenesis/Immune evasion
Q8NXH7	Glycine cleavage system H protein	6.64	Amino acid metabolism
A0A7H3W0W0	GTPase ObgE	6.64	Unknown/undefined
Q9FD87	HMG-CoA synthase	6.64	Lipid metabolism
A0A6M4II73	Hsp20/alpha crystallin family protein	6.64	Unknown/undefined
A0A2S6DKA1	Hydrolase	6.64	Pathogenesis/Adhesion
A0A7H3XLF1	Hydroxymethylglutaryl-CoA reductase, degradative	6.64	Lipid metabolism
A0A6M4IF77	Immunodominant staphylococcal antigen IsaB	6.64	Unknown/undefined
A0A389XS11	Immunoglobulin-binding protein Sbi	6.64	Pathogenesis/Immune evasion
A0A2I7Y9Q1	Iron-sulfur cluster carrier protein	6.64	Other
A0A7H3L1Q1	MAP domain-containing protein	6.64	Unknown/undefined
A0A380DRH8	N-acetylmuramoyl-L-alanine amidase, family 4	6.64	Other
A0A517IV59	Phage protein	6.64	Phage protein
A0A0E1X7A5	Probable cysteine desulfurase	6.64	Amino acid metabolism
A0A6G4Z5Z8	Probable glycine dehydrogenase (decarboxylating) subunit 1	6.64	Amino acid metabolism
A0A6K2KRN1	Proline dipeptidase	6.64	Amino acid metabolism
A0A6H3YKI1	PTS system glucose-specific transporter subunit IIA	6.64	Other
A0A6H3W6Q3	Queuine tRNA-ribosyltransferase	6.64	Nucleotide metabolism
A0A380E246	Queuosine Biosynthesis QueC ATPase	6.64	Other
A0A641ACK7	Reverse transcriptase-like protein	6.64	Other
A0A6H3YGY6	Ribosomal large subunit pseudouridine synthase D	6.64	Ribosome
A0A6K3Y1A2	Serine-aspartate repeat-containing protein D	6.64	Pathogenesis/Adhesion
A0A658X6S8	Signal recognition particle protein	6.64	Other
A6QIG6	Staphylococcal complement inhibitor	6.64	Pathogenesis /Immune evasion
A0A4T9ZRK6	Staphylokinase	6.64	Pathogenesis /Immune evasion
A0A679E9W0	Surface protein G	6.64	Unknown/undefined
A0A6K5WLP0	Tandem lipoprotein	6.64	Unknown/undefined
A0A7H3NAA5	Thiaminase II	6.64	Nucleotide metabolism
A0A7H9C3A1	Thimet oligopeptidase-like protein	6.64	Other
A0A2X2K372	tRNA-dihydrouridine synthase	6.64	Nucleotide metabolism
A0A2X2KDD8	Uncharacterized conserved protein	6.64	Unknown/undefined
A0A2X2K1R2	Uncharacterized conserved protein	6.64	Unknown/undefined
A0A0H2XJG9	Uncharacterized protein	6.64	Unknown/undefined
A0A145EYZ4	Uncharacterized protein	6.64	Unknown/undefined
A0A2S6D049	Uncharacterized protein	6.64	Unknown/undefined
A0A7H3T496	Uncharacterized protein	6.64	Unknown/undefined
A0A7H2HZJ5	Uncharacterized protein	6.64	Unknown/undefined
A0A6B0AQ14	Uncharacterized protein	6.64	Unknown/undefined
A0A0D1H2Z4	Uncharacterized protein	6.64	Unknown/undefined
UPI00019F4D2E	von Willebrand factor binding protein	6.64	Pathogenesis/Immune evasion
A0A6K4AD67	XRE family transcription regulator	6.64	Other
A0A7H2N2F0	YSIRK-type signal peptide-containing protein	6.64	Unknown/undefined
A0A7H4FTU9	Zinc metalloproteinase aureolysin	6.64	Pathogenesis/Protease
	*Proteins significantly more abundant in GTB/ST8 secretomes*		
P68824	Triosephosphate isomerase	6.49	Carbohydrate metabolism
A0A7H2IWP3	50S ribosomal protein L24	6.14	Ribosome
A0A6B5M3E9	Fructose-bisphosphate aldolase class 1	5.62	Carbohydrate metabolism
A0A6L0II03	Immunoglobulin G binding protein A	4.54	Pathogenesis /Immune evasion
A0A0H3JSF2	Enterotoxin P	4.54	Pathogenesis/Toxin
A0A0H3JP53	Uncharacterized protein	4.35	Unknown/undefined
A0A7H3VRD4	Uncharacterized protein	3.92	Unknown/undefined
A0A6B0BGX9	Class I SAM-dependent methyltransferase	3.21	Other
A0A6B3IRU7	EIIA-Lac	3.16	Carbohydrate metabolism
Q5HHM6	Extracellular matrix protein-binding protein emp	3.03	Pathogenesis/Adhesion
A0A659IFB3	Glutathione peroxidase	2.96	Other
A0A7H4DBP7	Bi-component leukocidin LukGH subunit H (LukH)	2.91	Pathogenesis/Cytolysis
A0A0E1VQM9	Galactose-6-phosphate isomerase subunit LacA	2.82	Carbohydrate metabolism
Q2YX95	Iron-regulated surface determinant protein A	2.75	Pathogenesis/Immune evasion
A0A0E8G970	ATL autolysin transcription regulator	2.75	Other
Q5HE16	6-phospho-beta-galactosidase	2.73	Carbohydrate metabolism
T1Y5M9	Phenol-soluble modulin alpha 4 peptide	2.73	Pathogenesis/Cytolysis
A0A6B5I4N5	D-lactate dehydrogenase	2.72	Carbohydrate metabolism
A0A0E1VKC6	Antibacterial protein 3	2.69	Pathogenesis/Cytolysis
A0A7H2FH39	50S ribosomal protein L33	2.69	Ribosome
A0A5F0HPC6	Tagatose 1,6-diphosphate aldolase	2.40	Carbohydrate metabolism
A0A7H3MQ17	HTH-type transcriptional regulator SarX	2.24	Pathogenesis/gene regulation
A0A2S6DFV8	Carbamate kinase	2.21	Aminoacid metabolism
A0A7H3MX82	YSIRK domain-containing triacylglycerol lipase Lip2/Geh	2.07	Lipid metabolism
A0A6H3WNU6	Serine hydroxymethyltransferase	2.05	Aminoacid metabolism
A0A0D1J8D5	Rhodanese	1.98	Other
A0A6C2A0Y6	Clumping factor B	1.94	Pathogenesis/Adhesion
A0A0H2XFP1	Type VII secretion system accessory factor EsaA	1.93	Pathogenesis/gene regulation
A0A7D8GGK5	Domain of uncharacterized function (DUF1963)	1.82	Unknown/undefined
A0A7H3CSS6	Hyaluronate lyase HysA	1.79	Carbohydrate metabolism
Q6G7C0	Galactose-6-phosphate isomerase subunit LacB	1.74	Carbohydrate metabolism
A0A0D1FNW3	30S ribosomal protein S11	1.74	Ribosome
A0A6H3XXE6	Tautomerase	1.66	Other
UPI0005C25D4C	Protein of unknown function DUF915	1.57	Unknown/undefined
T1Y9J1	Adenosine 5ʹ-monophosphoramidase	1.56	Other
A0A6B5EVI5	Elastin-binding protein EbpS	1.54	Pathogenesis/Adhesion
A0A0H3KH60	HTH-type transcriptional regulator rot	1.50	Pathogenesis/gene regulation
Q2FW51	Truncated MHC class II analog protein	1.50	Pathogenesis/Toxin

**Table 2.** Lists of the 68 proteins found exclusively (log_2_ − 6.64) or at significantly higher levels (log_2_ > −1.50) in the secretome of GTS/ST398 strains grown in brain-heart infusion (BHI) broth. The information reported in the first three columns (accession number, protein name, and abundance ratio – AR – expressed as the log_2_ value) was obtained by Proteome Discoverer Analysis. The last column reports biological process and molecular function information as found in the UniProtKB. The definition “other” indicates that the protein belongs to metabolic pathways different than amino acid, carbohydrate, lipid, or nucleotide metabolism

Accession	Description	AR (log_2_)		Process/function	
	*Proteins detected only in GTS/ST398 secretomes*				
A0A0E0VMF5	3Beta_HSD domain-containing protein	−6.64		Lipid metabolism	
A0A2S6DH91	ABC transporter ATP-binding protein	−6.64		Other	
A0A2S6DRW7	Amidase domain-containing protein	−6.64		Other	
C4B4S0	Coagulase	−6.64		Pathogenesis/Immune evasion	
A0A2X2K0V2	D-arabino-3-hexulose 6-phosphate formaldehyde lyase	−6.64		Nucleotide metabolism	
A0A0E1XAV7	DNA-binding helix-turn-helix protein	−6.64		Phage	
UPI0013F1281B	Protein of unknown function DUF4889	−6.64		Unknown	
A0A6B5D256	DUF5085 family protein	−6.64		Unknown	
A0A499S7K4	Enterotoxin SER	−6.64		Pathogenesis/Toxin	
A0A0E0VL58	Exotoxin	−6.64		Pathogenesis/Toxin	
A0A7H2N7L7	Fibronectin-binding protein FnbB	−6.64		Pathogenesis /Adhesion	
A0A4T9Z0G4	Flavohemoglobin	−6.64		Other	
A0A1C8Y884	Gamma-hemolysin component C	−6.64		Pathogenesis/Cytolysis	
A0A7D8CBJ6	Glycerol phosphate lipoteichoic acid synthase	−6.64		Lipid metabolism	
A0A380DYG8	Glycerol-3-phosphate dehydrogenase	−6.64		Lipid metabolism	
A0A5C8X2J5	Hsp70 family protein	−6.64		Other	
A0A7H4CA57	Ig domain-containing protein	−6.64		Phage	
A0A5C8X7X3	Isocitrate dehydrogenase [NADP]	−6.64		Lipid metabolism	
A0A2X2K931	Lipoprotein, putative	−6.64		Unknown	
A0A6M1X978	Magnesium transporter	−6.64		Other	
A0A1Q8DGJ8	Mannitol-1-phosphate 5-dehydrogenase	−6.64		Carbohydrate metabolism	
Q2UWP2	MHC class II analog protein (Map)	−6.64		Pathogenesis/Adhesion	
A0A7H2N323	MSCRAMM family adhesin clumping factor ClfA	−6.64		Pathogenesis/Adhesion	
A0A7H9CBC0	Major capsid protein	−6.64		Phage	
A0A6K8HCR0	Phospholipase C/beta-hemolysin	−6.64		Pathogenesis/Cytolysis	
A0A6B5HY23	Polysaccharide lyase 8 family protein	−6.64		Other	
A0A0E0VL93	Putative endopeptidase lytE	−6.64		Other	
A0A7D5TP35	Putative lipoprotein	−6.64		Phage	
A0A6N3YL97	Restriction endonuclease	−6.64		Other	
D7RM10	Translation elongation factor Tu	−6.64		Other	
A0A2S6DP62	HesA/moeB/thiF family protein	−6.64		Other	
A0A2S6DEW1	DNA-binding protein	−6.64		Other	
A0A7H3PU96	Uncharacterized protein	−6.64		Phage	
A0A7H3IWE4	Uncharacterized protein	−6.64		Unknown	
A0A6B0BAG1	Uncharacterized protein	−6.64		Phage	
A0A6H3YUP1	Uncharacterized protein	−6.64		Unknown	
A0A6K0L0H3	XkdX family protein	−6.64		Phage	
	*Proteins significantly more abundant in GTS/ST398 secretomes*				
A0A2S6DHX1	Phospholipase C /Beta-hemolysin	−5.88		Pathogenesis/Cytolysis	
A0A5C8X3K0	Glutamyl endopeptidase (SspA)	−4.94		Pathogenesis/Protease	
A0A0J9X1Z2	Alpha-hemolysin	−4.23		Pathogenesis/Cytolysis	
UPI00002322F9	Staphopain B (SspB)	−4.09		Pathogenesis/Protease	
A0A454GWS5	Alpha-hemolysin	−3.86		Pathogenesis/Cytolysis	
A0A0E0VTT6	Neutral metalloproteinase	−3.82		Pathogenesis/Protease	
A0A5S9I5Q8	Triacylglycerol lipase	−3.78		Lipid metabolism	
A0A229LUA6	HlyD family efflux transporter periplasmic adaptor subunit	−3.68		Pathogenesis/multidrug resistance	
A0A2S6DP88	Leukocidin S subunit	−3.19		Pathogenesis/Cytolysis	
A0A7D8GH46	Gamma-hemolysin component B	−3.04		Pathogenesis/Cytolysis	
A0A4T9ZIV1	Bi-component leukocidin LukGH subunit G	−2.78		Pathogenesis/Cytolysis	
A0A0E0VR87	Outer membrane protein	−2.61		Unknown	
A0A6B5I402	Fibrinogen-binding protein	−2.46		Pathogenesis/Adhesion	
Q2YY67	L-threonine dehydratase catabolic TdcB	−2.45		Amino acid metabolism	
A0A6B5CIZ8	Phage major capsid protein	−2.31		Phage	
P0A0M2	Delta-hemolysin	−1.93		Pathogenesis/Cytolysis	
A0A7H2N4Z9	Alanine dehydrogenase	−1.92		Amino acid metabolism	
A0A0E0VP07	Uncharacterized protein	−1.90		Unknown	
Q9AFA9	Leukocidin LukS component	−1.82		Pathogenesis/Cytolysis	
A0A6A9GX73	Uncharacterized protein	−1.77		Unknown	
A0A0H3JW27	MW1057 protein	−1.76		Pathogenesis/Cytolysis	
A0A6B0AT46	MSCRAMM family adhesin SdrE	−1.75		Pathogenesis/Adhesion	
W8UVT0	Alkaline shock response membrane anchor protein AmaP	−1.66		Other	
A0A0U1MXM6	NAD(P)H-binding protein	−1.64		Lipid metabolism	
A0A0E0VTR1	Leukocidin F subunit	−1.63		Pathogenesis/Cytolysis	
A0A7H3UQ41	Bifunctional autolysin	−1.58		Other	
A0A0E0VP14	Micrococcal nuclease	−1.56		Pathogenesis /Nuclease	
A0A0E1VJY8	DM13 domain-containing protein	−1.54		Unknown	
A0A0E0VMJ2	Putative exported protein	−1.51		Unknown

**Figure 5.**
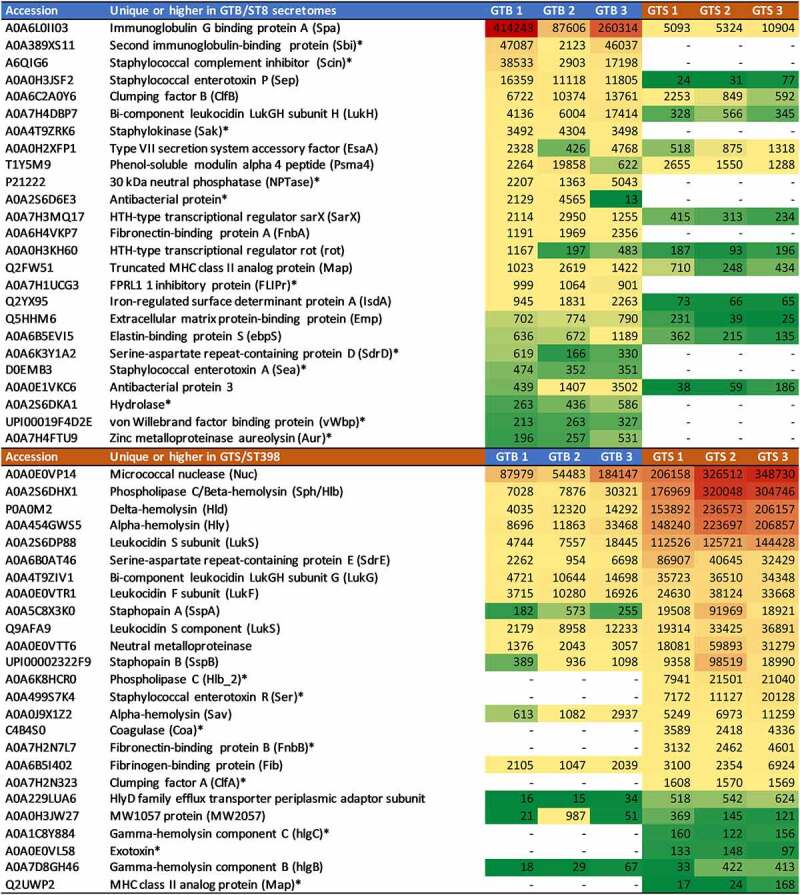
Heatmap of the extracellular virulence factors showing significant differences between the two GT/ST, reported in order of abundance in the respective GT/ST group. The first and second columns report the protein accession number and the protein name and acronym. The last six columns illustrate in a heat map the average normalized protein abundance value/1000 calculated for each strain with Proteome Discoverer. Color intensity ranges from the highest observed value (dark red) to the lowest observed value (dark green). White: the protein was not detected. The proteins detected only in one genotype are marked with an asterisk.

**Figure 6.**
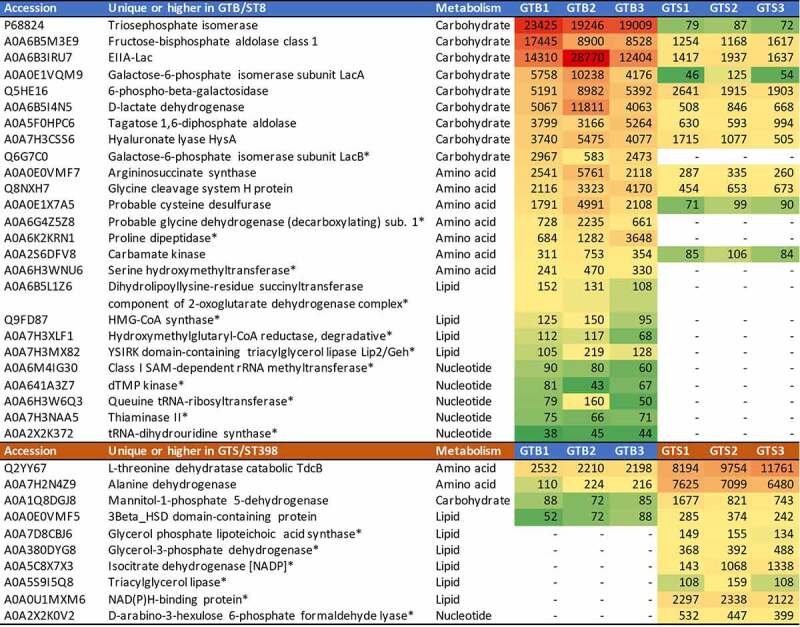
Heatmap of the extracellular metabolic enzymes showing significant differences between the two GT/ST, reported in order of abundance in the respective GT/ST group. The first and second columns report the protein accession number and the protein name and acronym. The third column indicates the metabolic pathway. The last six columns illustrate in a heat map the average normalized protein abundance value/1000 calculated for each strain with Proteome Discoverer. Color intensity ranges from the highest observed value (dark red) to the lowest observed value (dark green). White: the protein was not detected. The proteins detected only in one genotype are marked with an asterisk.

The differential extracellular virulence factors (those reporting the term “pathogenesis” in their UniProtKB entry page as reported in Tables 1 and 2) and metabolic enzymes (also according to UniProtKB as reported in Tables 1 and 2) were arranged in heatmaps for the six investigated strains according to the genotype and to the normalized protein abundance value. The virulence factor heatmap is reported in Figure 5 and the extracellular metabolic enzyme heatmap is reported in Figure 6.

Figure 5 highlights the crucial differences in terms of identity and abundance of virulence factors released by the two strain groups. In GTB/ST8, the differential proteins with the highest abundances were Spa, Sbi and Scin, all mediating the evasion of innate and adaptive humoral immunity. Notably, the last two were not identified in the GTS/ST398 supernatants. On the other hand, the differential protein predominating in the GTS/ST398 supernatants was Nuc. This protein, however, was highly abundant also in the GTB/ST8 secretomes according to its normalized protein abundance value (the second in order of abundance after Spa). This was followed by leukocidins and hemolysins, mediating evasion of cellular immunity, adhesins and proteases. Another remarkable difference was the type of coagulase detected in the two genotypes, as GTB/ST8 secreted the von Willebrand factor while GTS/ST398 secreted the classical coagulase. The normalized protein abundance values of the two proteins were also quite different (about one order of magnitude).

Figure 6 reports the extracellular metabolic enzymes showing statistically significant differences in the two genotypes, with their respective normalized protein abundances. Metabolic enzymes were significantly more abundant in the GTB/ST8 than in the GTS/ST398 secretome (26 *vs* 10, respectively). Carbohydrate metabolism enzymes were the most represented, and almost all of them were consistently more abundant in the GTB/ST8 secretome (9 *vs* 1). The glycolytic enzyme triose phosphate isomerase was the highest, followed by another glycolytic enzyme, fructose-bisphosphate aldolase. Lactose and galactose metabolism enzymes were next. Carbohydrate metabolism enzymes were followed by aminoacid metabolism enzymes, also more represented and abundant in the GTB/ST8 secretome than in the GTS/ST398 secretome (7 v*s* 2). Nucleotide metabolism enzymes followed the same behavior. On the other hand, lipid metabolism enzymes were more represented and abundant in the GTS/ST398 than in the GT8/ST8 secretome (6 *vs* 4, respectively).

### Cell viability assay

**Figure 7.**
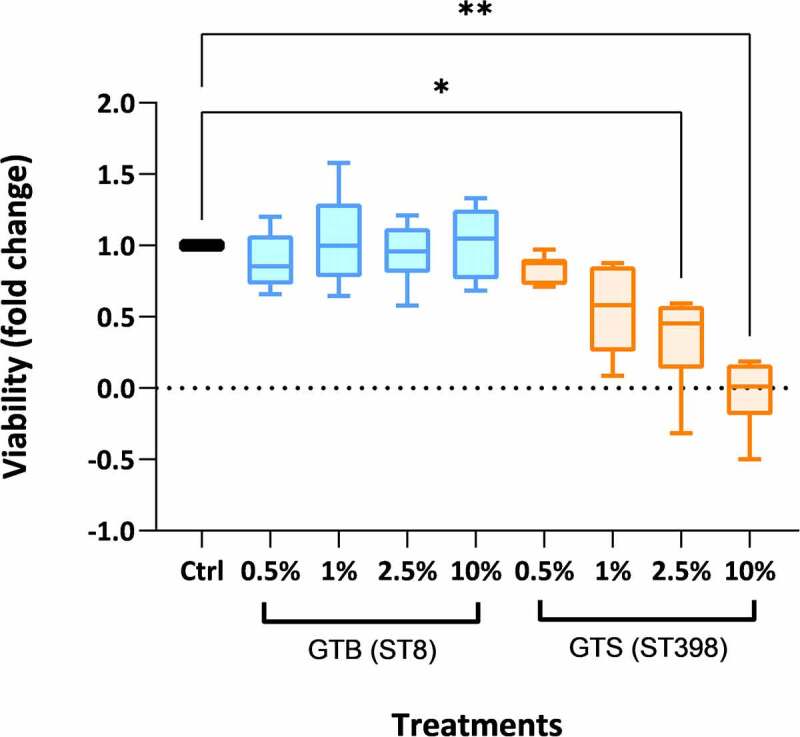
Peripheral blood mononuclear cell (PBMC) viability after 18 h of incubation with the proteins secreted in brain-heart infusion (BHI) broth by the two *Staphylococcus aureus* GT (ST) evaluated in this work. The viability is expressed as fold-change compared to cells incubated without secreted bacterial proteins (control) in six technical replicates per condition. Significance was accepted at P < 0.05 (*) and P < 0.01 (**). The lines inside the boxes denote the median. The whiskers indicate the variability outside the upper and lower quartiles.

To evaluate the effect of GTB/ST8 and GTS/ST398 secretomes on cell viability, an MTT test was performed by incubating bovine PBMCs for 18 hours with the proteins secreted by the two genotypes at different concentrations. The proteins secreted in BHI by the GTS/ST398 and by the GTB/ST8 strains had a concentration of 10 and 8 µg/mL, respectively, and were used at a dilution of 0.5%, 1%, 2.5%, and 10% (Figure 7). After 18 hours, the PBMCs incubated with 2.5% and 10% dilutions of the GTS/ST398 secretions showed a significant decrease in viability compared to the control (P < 0.05 and P < 0.005, respectively, calculated on six technical replicates per condition). On the other hand, no significant differences with the control were observed with the GTB/ST8 secretions up to the highest concentration tested.

## Discussion

GTB/ST8 *S. aureus* are associated with a very high within-herd prevalence as opposite to other genotypes including GTS/ST398 [6,24]. Strain diffusivity is a crucial issue in herds infected by *S. aureus*, because knowing the potential behavior of the strain responsible for the mastitis outbreak might prompt different intervention approaches. Moreover, the secreted proteins most associated with strain diffusivity and persistence may represent important future targets for diagnosis and prevention. Therefore, the aims of this study extend well beyond these two model GT/ST. A previous article compared the genome and transcriptome of six *S. aureus* strains belonging to the two genotypes to shed light on the relationships of virulence gene carriage and expression with epidemiological behavior [9]. Here, we extended our investigation to the secretome by applying high-performance shotgun proteomics to further examine their virulence phenotypes. We found numerous and relevant differences, in accordance with other authors’ reports that *S. aureus* genotypes can have very diverse secretomes, which are related to their pathogenetic behavior [16,17,25].

The most abundant differential proteins in the GTB/ST8 secretomes were the immunoglobulin G binding protein A (Spa), followed by the immunoglobulin-binding protein (Sbi) and the staphylococcal complement inhibitor (Scin). Spa protects *S. aureus* from antibody-mediated phagocytic killing with its ability to capture both the Fc and Fab region of immunoglobulins [26]. The Sbi protein, a multifunctional immune evasion factor of *S. aureus* [27], can bind IgG similarly to protein A as well as C3, promoting its unproductive consumption in the fluid phase and acting as a potent complement inhibitor of the alternative pathway-mediated lysis [27]. Scin inhibits all three complement pathways: the alternative, classical, and lectin pathways [26]. By inactivating C3 convertases, it blocks most complement functions including opsonization, phagocytosis, and neutrophil killing [13]. The regulation appears to occur also at a transcriptomic level, as in our previous study Scin was over-expressed in GTB/ST8 and down-regulated in GTS/ST398 [9].

Early expression of Scin together with chemotaxis-inhibitory proteins drives instant immune evasion [28]; we detected FPRL-1, a protein of the CHIPS-FLIPr family, only in GTB/ST8. Another protein found only in GTB/ST8 was the 30 kDa neutral phosphatase, a highly cationic enzyme capable of binding immunoglobulins and serum albumin. *S. aureus* is also equipped with virulence factors that target complement without direct binding to C3 convertase. Staphylokinase (Sak) was detected only in the GTB/ST8 secretome. Sak is a secreted protein that binds plasminogen converting it into its active form plasmin [29]. Plasmin, a serine protease, is bound externally to *S. aureus* and degrades C3 convertase-dependent C3b to prevent deposition on the bacterial surface. In light of the limited functionality of the classical pathway in milk [30], the ability of GTB/ST8 to target both the adaptive and innate arms of the complement may represent an advantage in terms of immune evasion capabilities in the mammary gland.

Another crucial defense of the mammary gland against intramammary infection are antimicrobial peptides and proteins, including defensins and cathelicidins [31,32], released by both neutrophils and mammary epithelial cells [33,34]. In response, *S. aureus* secretes many proteins aimed at neutralizing them. Sak and aureolysin (Aur) have essential roles in binding defensin peptides and in cleaving and inactivating cathelicidins, respectively [35]. Notably, cathelicidins are among the few antibacterial peptides with potent anti-staphylococcal activity; thus, *S. aureus* strains producing these two proteins are significantly more resistant to cathelicidins than Aur-negative strains [36]. In our study, both Sak and Aur were found uniquely in the secretome of GTB/ST8 strains. The iron-regulated surface determinant protein A (IsdA) was also higher in their secretome. IsdA is a cell wall-anchored surface receptor that protects *S. aureus* against the bactericidal protease activity of apolactoferrin and bovine lactoferricin, relevant host defense mechanisms against bacterial infection in milk [37]. IsdA also plays a crucial role in immune evasion by enhancing bacterial cellular hydrophobicity, thereby increasing the resistance of *S. aureus* to beta-defensins and cathelicidins [35]. The presence of redundant mechanisms aimed at degrading and neutralizing antimicrobial peptides and proteins translates into a significant advantage to GTB/ST8 in terms of persistence in the mammary gland.

Metabolic enzymes, especially those belonging to the carbohydrate metabolism, were significantly more abundant in the secretome of GTB/ST8 strains. Enzymes degrading lactose and galactose may advantage bacterial growth in milk; extracellular metabolic enzymes, however, can have a quite more relevant role in the host/bacterium relationship. Mekonnen and coworkers [38] observed that signatures of cytoplasmic proteins in the secretome represent a distinguishing feature of genotypes with different epidemiologic behavior and intracellular survival capabilities. According to these authors, cytoplasmic proteins liberated in the extracellular milieu contribute substantially to staphylococcal virulence by playing moonlighting and immune evasion functions [39]. The concept of moonlighting proteins is one in which a single protein with alternative oligomeric conformations can carry out different functions when located inside or outside the cell. Several authors now agree that moonlighting proteins are crucial for *S. aureus* pathogenicity [40]. Further, several moonlighting proteins can play multiple roles in different infection stages, thus enhancing the virulence of the bacterium [40]. A relevant advantage of moonlighting proteins is their better ability to hide from the host immune system, as these typically exhibit high structural conservation toward their host counterparts. Accordingly, proteins involved in critical metabolic pathways and ancestral processes, including ribosomal proteins, molecular chaperones, and glycolytic enzymes, typically exhibit moonlighting activities. In line with this, we also detected several ribosomal proteins only in the GTB/ST8 secretome. The relevance of carbohydrate metabolism enzymes in moonlighting and immune evasion is highlighted by their presence in the secretome of both genotypes, albeit at consistently higher levels in GTB/ST8 than GTS/ST398.

On the other hand, almost all the hemolysins and leukocidins detected in this work were significantly more abundant in the GTS/ST398 secretome, in agreement with the previous genomic and transcriptomic characterization of the same strains [9]. These included alpha, delta, beta, and gamma hemolysins, and various leukocidins. All these proteins target and kill leukocytes, the primary cellular defense of the mammary gland, by destabilizing their membrane or by forming pores leading to osmotic lysis [41]. In addition, many hemolysins and pore-forming proteins also exploit cellular pathways to enhance cell killing, including the inflammasome pathway. For example, staphylococcal leukocidins activate the NOD-, LRR- and pyrin domain-containing protein 3 (NLRP3) inflammasome in macrophages and monocytes, potentiating lysis and leading to pyroptosis with enhanced production of pro-inflammatory cytokines [41].

The associations between a higher abundance of cytotoxic proteins [including hemolysins and leukocidins) in GTS/ST398 and a higher cytotoxic/hemolytic potential were validated with an *in vitro* viability assay on bovine PBMCs. In fact, contrasting transcriptomic data had been obtained by Capra and coworkers [9], who hypothesized a higher potential cytotoxicity of GTB/ST8 strains. In addition, it was shown earlier [42] that alpha-hemolysin could be degraded by staphopain A (SspA), both proteins found in large amounts in GTS/ST398. Thus, the net effect could not be extrapolated even under the used growth conditions in BHI, and it was experimentally validated. The PBMC viability assay carried out in this work clearly showed that the observed protein abundance differences do indeed translate into a higher cytotoxic potential of the GTS/ST398 strains when compared to the GTB/ST8 strains. Strikingly, the latter did not produce visible effects on cell viability even after 18 h of incubation at the highest tested concentration.

Bi-component leukocidins consist of two separately secreted components, named S and F based on their elution by cationic exchange chromatography (slow *vs* fast, respectively) [41]. The binding of S component to the cell membrane is required for the secondary binding of F component, leading to the formation of membrane pores and cellular lysis [43]. In the LukGH (or LukAB), LukG is the S component, while LukH is the F component. LukH was the only leukocidin that increased in the GTB/ST8 secretomes. Accordingly, the higher abundance of LukH in GTB/ST8 should not increase the cytotoxic capabilities of this genotype, while in GTS/ST398 LukG may engage the LukF component to form heterocomplexes. All this considered, cows infected by strains behaving as GTS/ST398 might be at higher risk for developing clinical mastitis.

The GTS/ST398 secretomes were also significantly higher in extracellular proteases, including SspA and SspB and the neutral metalloprotease. Staphopains are proteases with broad specificity implicated in tissue colonization and connective tissue destruction and may act as an immune evasion factor by cleaving immunoglobulins and complement components. Staphopain A is required for proteolytic maturation of SspB, and both are involved in the inhibition of neutrophil recruitment and activation. Staphopain B also bocks phagocytosis of opsonized *S. aureus* by neutrophils and monocytes by inducing their death in a proteolytic activity-dependent manner [44,45]. Higher expression of staphopains in GTS/ST398 strains was also detected by transcriptomics [9]. However, it should be noted that a high amount of SspA (as found in GTS/ST398) does not necessarily correlate with higher proteolytic activity, since the zymogen SspA needs to be exclusively activated by Aur [46,47], which was found in much higher amounts in GTB/ST8. Thus, even though GTB/ST8 strains exhibit lower amounts of SspA, substantial activation by Aur could finally lead to a higher proteolytic activity of GTB/ST8 compared to GTS/ST398.

One of the most abundant proteins found in the secretome of both genotypes, with significantly higher amounts in GTS/ST398, was the micrococcal nuclease (Nuc). This enzyme facilitates bacterial escape from neutrophil extracellular traps (NETs) [48,49]. NETs are structures composed of DNA, histones, and antimicrobial proteins that are released extracellularly by neutrophils as a means for trapping and killing invading pathogens [50]. NETs represent a crucial defense against mammary gland pathogens [22,51], and the finding of Nuc as one of the most abundant secreted proteins in both genotypes underlines its relevance for bacterial virulence. Leukocidins can potentiate NET formation, exacerbating the inflammatory response [52] and favoring the onset of clinical mastitis. Furthermore, fibronectin-binding proteins (Fnb) A was higher in GTB/ST8, while FnbB was higher in GTS/ST398. Fnbs are cell wall proteins possessing fibronectin, fibrinogen, and elastin-binding regions, but FnbB also confers resistance to the bactericidal activity of NETs. Intriguingly, in some specific contexts or tissues *S. aureus* and other pathogens may benefit more from inducing NETs and using them to damage host tissues, together with secreted proteases, than from blocking or avoiding their activation [49,53,54]. This strategy can provide better access to metabolic resources, favor deeper tissue colonization, and ensure safer and optimal survival, and might enable GTS/ST398 strains to colonize different tissues and hosts. Conversely, in the case of GTB/ST8 strains, evasion of the immune response through inflammation-dampening mechanisms including moonlighting and molecular mimicry, Ig binding, complement inhibition and antimicrobial peptide neutralization, might represent a better “escape strategy” in the specific context of the mammary gland, enabling these strains to establish chronic, subclinical infections, and infect a higher number of animals in the herd. As type 3 immunity is probably the most relevant defense mechanism in the mammary gland [55], a higher ability to avoid inflammation might translate into better chances for maintaining and spreading infection.

Strikingly, the staphylococcal coagulase was detected only in GTS/ST398 secretions. Coagulase (Coa) is an enzyme that specifically forms a complex with prothrombin and can clot fibrinogen without any proteolytic cleavage. The ability to clot fibrinogen is so typical that it is used in the microbiology laboratory to discriminate *S. aureus* from almost all other staphylococcal species, and it constitutes a crucial immune evasion strategy of this pathogen [35]. Yet, plasma coagulation can be mediated by other *S. aureus* proteins, including the von Willebrand factor binding protein (vWbp) and Sak [56] detected only in GTB/ST8. Therefore, we might speculate that proteins with different coagulase properties released by the GTB/ST8 strains, together with other proteins or processes to be identified, may render them more capable of acting on the high-abundance proteins typical of this host niche, in which fibrinogen and plasminogen are scarce but milk whey proteins are largely available. The differential abundances of clumping factor A (higher in GTS/ST398) and clumping factor B (higher in GTB/ST8) may also have a role in their adaptation to different host niches, as their binding specificities differ [57]. Dedicated biochemical studies will be required to clarify these aspects. Notably, however, all GTB/ST8 strains coagulated milk whey proteins while growing in this medium, while GTS/ST398 did not.

Enterotoxins also showed relevant differences. Enterotoxin Sep was more abundant, and enterotoxin Sea was uniquely found in GTB/ST8 secretions. On the other hand, enterotoxin Ser was found uniquely in GTS secretions. Both observations were in line with genomic and transcriptomic studies [9]. Acting as superantigens in the mammary gland, enterotoxins massively activate T lymphocytes and antigen-presenting cells, interfering with the generation of a proper adaptive immune response [35].

Mekonnen^38^ observed that distinctive features in the bacterial secretomes associated with virulence were related primarily to the accessory genome. Phage-encoded proteins predominated in GTS/ST398 secretomes, reinforcing the notion that these mobile genetic elements play a relevant role in modulating *S. aureus* pathogenesis [58].

Major differences in the composition of *S. aureus* proteomes are related to differences in transcriptional regulation by the *agr* system, resulting in the expression of diverse secreted virulence factors [17]. Indeed, the comparative secretome analyses of dominant human- or livestock-associated lineages of ST8 and ST398 revealed that specific virulence factors are differentially secreted because of regulatory differences linked to *agr* activities [16]. In this study, we found significantly higher amounts of the HTH-type transcriptional regulator SarX in GTB strains. This protein is involved in the regulation of virulence genes by binding directly to the *agr* promoter region and acting as a repressor of the *agr* locus. It consequently targets the genes regulated by the *agr* system such as *sspA, hla* and *hlb*. Furthermore, the HTH-type transcriptional regulator rot was also higher in GTB. This is a global regulator with both positive and negative effects that mediates modulation of several genes involved in virulence. GTB/ST8 showed a higher expression of Target of RNAIII activating Protein (TRAP) that leads to the activation of *agr* [9]. Accordingly, the phenotypic differences seen in the two secretomes could be related to the differential expression of these critical transcriptional regulators that might be crucial in host adaptation. However, this will need to be investigated further with dedicated molecular approaches.

However, a limitation of this study lies in the generation of secreted proteins through bacterial culture in a conventional laboratory medium. We analyzed the secretome at 3.5 h of culture, in the exponential growth phase, but other secretome differences may emerge if analyzed at other time points. Adding to this, the presence of milk proteins and bacterial inhibitors, the interaction with host cells, and other stimuli provided by the *in vivo* environment, including other staphylococcal species, might influence the nature and relative levels of secreted proteins. Yet, applying proteomics to complex fluids such as milk or milk whey still poses tremendous challenges related to the massive amounts of caseins and high-abundance whey proteins that would severely hamper the detection of secreted *S. aureus* proteins. On the other hand, cell culture models introduce variables associated with the presence of eukaryotic cell proteins, other components of the growth medium, and bacterial internalization or cell invasion. This notwithstanding, further *in vitro* and *in vivo* investigations will be crucial for understanding the role of the bacterial secretome in host-pathogen interactions. Furthermore, strain-specific differences within a genotype can be present that will need to be considered.

## Final considerations and conclusion

The transcriptomic comparison of the strains investigated here revealed the functional enrichment of genes related to adaptation and chronicity in GTB/ST8 *versus* GTS/ST398 *S. aureus* [9]. In the present study, we confirmed and expanded those findings by observing the preferential release by GTB/ST8 of virulence factors favoring the establishment of chronic, subclinical infections with immune-dampening activities and a higher ability to evade both innate and adaptive humoral responses, versus a higher propensity of GTS/ST398 for establishing acute infections with pro-inflammatory activities, neutrophil killing, NETosis, and pyroptosis. We observed significant differences in the expression and secretion of crucial virulence genes present in both genotypes, such as leukocidins, hemolysins, proteases, complement-binding and immunoglobulin-binding proteins, as well as metabolic enzymes. Therefore, investigating gene carriage alone, although crucial for understanding strain circulation and virulence potential, is likely not sufficient for establishing meaningful correlations with the epidemiological behavior or clinical severity of a particular strain. As recently concluded in a large study investigating the correlation of virulence gene carriage with the clinical outcome, it is differential gene expression (and secretion) rather than gene carriage that affects the clinical presentation of IMI [24]. Accordingly, investigating in more detail the secreted virulence factor characteristics will also help to unravel some controversial aspects concerning the role of cytotoxicity in *S. aureus* pathogenicity in bovine mastitis in general, and for GTS/ST398 in particular, as conflicting results are published [3,6,59–62].

Such an impressive heterogeneity in the secretome of the two investigated *S. aureus* genotypes requires consideration also for its implications for mastitis control and prevention. So far, the development of an effective vaccine for *S. aureus* mastitis has encountered many difficulties. As nicely discussed recently by de Jong and coworkers [26], this can be attributed to several factors, including the extreme variability of the bacterial surfaceome and exoproteome. Accordingly, vaccine strategies implementing single antigens without adjuvant have not been successful in providing protection. Future vaccine efforts should incorporate a combination of proteins, including evasion molecules, and raise antibodies against them. Nevertheless, the redundancy and multiplicity of immune evasion strategies, as clearly emerged also in this study, remains a challenge [26]. We will also need to understand in better detail the roles of humoral immunity, innate immunity, tolerance, type 3 immunity, and the microbiota in the mammary gland response to infection, as well as the complex kinetics and interactions of immune evasion factors with host factors [55,63,64,65–66]. Still, understanding the complexities in the *S. aureus* secretome will be a crucial step toward this goal.

## Data Availability

The mass spectrometry proteomics data have been deposited to the ProteomeXchange Consortium [65] via the PRIDE partner repository [66] with the dataset identifier PXD029571.
